# Hybrid fiber optic-fMRI for multimodal cell-specific recording and manipulation of neural activity in rodents

**DOI:** 10.1117/1.NPh.9.3.032206

**Published:** 2022-03-21

**Authors:** Horea-Ioan Ioanas, Felix Schlegel, Zhiva Skachokova, Aileen Schroeter, Tetiana Husak, Markus Rudin

**Affiliations:** a University of Zurich Institute for Biomedical Engineering, ETH, Zürich, Switzerland; b Massachusetts Institute of Technology, Department of Biological Engineering, Cambridge, Massachusetts, United States; c Dartmouth College, Center for Open Neuroscience, Hanover, New Hampshire, United States; d University of Zurich, USZ Innovation Hub, Zurich, Switzerland; e Massachusetts Institute of Technology, Department of Electrical Engineering and Computer Science, Cambridge, Massachusetts, United States; f The LOOP Zurich, Zurich, Switzerland

**Keywords:** optics, functional magnetic resonance imaging, multimodal, neuroimaging, optogenetics, neuroscience, technology

## Abstract

**Significance:**

Multiscale imaging holds particular relevance to neuroscience, where it helps integrate the cellular and molecular biological scale, which is most accessible to interventions, with holistic organ-level evaluations, most relevant with respect to function. Being inextricably interdisciplinary, multiscale imaging benefits substantially from incremental technology adoption, and a detailed overview of the state-of-the-art is vital to an informed application of imaging methods.

**Aim:**

In this article, we lay out the background and methodological aspects of multimodal approaches combining functional magnetic resonance imaging (fMRI) with simultaneous optical measurement or stimulation.

**Approach:**

We focus on optical techniques as these allow, in conjunction with genetically encoded proteins (e.g. calcium indicators or optical signal transducers), unprecedented read-out and control specificity for individual cell-types during fMRI experiments, while leveraging non-interfering modalities.

**Results:**

A variety of different solutions for optical/fMRI methods has been reported ranging from bulk fluorescence recordings via fiber photometry to high resolution microscopy. In particular, the plethora of optogenetic tools has enabled the transformation of stimulus-evoked fMRI into a cell biological interrogation method. We discuss the capabilities and limitations of these genetically encoded molecular tools in the study of brain phenomena of great methodological and neuropsychiatric interest—such as neurovascular coupling (NVC) and neuronal network mapping. We provide a methodological description of this interdisciplinary field of study, and focus in particular on the limitations of the widely used blood oxygen level dependent (BOLD) signal and how multimodal readouts can shed light on the contributions arising from neurons, astrocytes, or the vasculature.

**Conclusion:**

We conclude that information from multiple signaling pathways must be incorporated in future forward models of the BOLD response to prevent erroneous conclusions when using fMRI as a surrogate measure for neural activity. Further, we highlight the potential of direct neuronal stimulation via genetically defined brain networks towards advancing neurophysiological understanding and better estimating effective connectivity.

## 1 Introduction

Noninvasive functional brain imaging has made significant contributions to the understanding of mammalian brain functional architecture and of its adaptation to drug interventions and changes imposed by pathological conditions. Among such imaging modalities, blood-oxygen-level-dependent functional magnetic resonance imaging (BOLD fMRI) is probably the most widely used surrogate measure of neural activity in humans.^1^^–^^3^ The method is attractive as it is noninvasive, does not require the administration of contrast agents, is fully three-dimensional (3D), and can provide whole brain coverage. The contrast mechanism underlying the BOLD signal is based on activation-induced changes in the oxygen extraction from blood, which affects the ratio of diamagnetic oxygenated (Hb) to paramagnetic deoxygenated hemoglobin (dHb), with dHb acting as an intrinsic MRI contrast agent. The resulting local change in magnetic susceptibility affects the transverse relaxation rates ($R_{2}$, $R_{2}*$), which leads to changes in signal intensity if $R_{2}$ (spin echo) or $R_{2}*$-sensitive (gradient echo) fMRI acquisition protocols are being used. Alternatively, activation-induced changes in cerebral blood flow (CBF) or blood volume (CBV) can be assessed using fMRI methods.^4^ Despite these obvious advantages, it is important to realize that fMRI-based functional readouts measure hemodynamic alterations elicited by neural activity and therefore constitute an indirect measure of brain activity. The validity of the surrogate depends on the integrity of the neurovascular (and neurometabolic) coupling (NVC), i.e., the link between neural activity and the local hemodynamic adaptation. In fact, this is the case for many functional imaging methods assessing brain activity, such as intrinsic optical imaging,^5^ near-infrared spectroscopic (NIRS) imaging,^6^ label-free photoacoustic imaging,^7^ and ultrasound imaging.^8^ Sophisticated biophysical models have been developed to account for the dynamic behavior of the BOLD fMRI signal,^9^^–^^11^ capable of describing BOLD responses in a semiquantitative manner. In these models, complex biological processes such as the triggering of the vasodilatory signal by neuronal activity are lumped into single parameters and thus do not provide insight into the cellular and molecular mechanisms responsible for functional hyperemia (FH). However, detailed knowledge of the mechanisms underlying neurometabolic and NVC is essential to understand the spatiotemporal behavior of functional signals and to correctly interpret fMRI responses, in particular under pathological conditions or when assessing responses to therapeutic interventions. Apart from this basic question on the nature of the BOLD fMRI signal, deciphering the role of specific cell populations in information processing is essential for elucidating the underpinnings of brain networks, i.e., analyzing how specific cell-types affects brain wide functional responses.

Animal models play an important role in addressing these questions. While most of the pioneering fMRI research has been performed in primates and rats,^12^^,^^13^ improvements in MR sensitivity have made it possible to study animals such as mice. With the mouse being a preferred organism in genetic and molecular biological applications, fMRI is now compatible with a plethora of both invasive and noninvasive tools capable of providing highly specific information on the behavior of specific cell types and the molecular actors mediating it. For example, *in vivo* microscopy techniques allow the deconvolution of macroscopically lumped FH effects into contributions of individual tissue components (e.g., microvascular segments) resolved in space and time.^14^

Further research^15^^,^^16^ has suggested the development of bottom-up forward models linking sub-population-specific neuronal activity to experimentally accessible responses in various microvascular segments. Such research would integrate cell-type-specific activity into the FH response derived from noninvasive imaging (with FH determining the change in BOLD contrast), thereby “bridging [the gap] from microscopic to macroscopic scales.” While this conceptual framework is convincing and lays out a clear path for the elucidation of the neurovascular response, it faces substantial challenges. Local microvascular responses may not be easily translated to FH as observed by noninvasive imaging, since the irrigation volume of an “active” brain region may extend beyond the field-of-view of microscopy techniques, and effects occurring outside the field of view (FOV) would also influence the FH. Hence, information should be collected across scales and integrated across space and time to match the dimensions accessible by noninvasive imaging methods (e.g., Ref. 17). Moreover, the application of extant *in vivo* microscopy techniques to the study of deep brain structures may become challenging.

Alternatively, information on the contribution of individual cell-types to FH may be obtained using cell-specific labeling strategies in combination with an imaging readout that collects data from a volume comparable to that of the noninvasive fMRI imaging method. The hybrid setup can be designed such that multiple signals can be sampled simultaneously, which becomes critical under conditions in which scan-to-scan variability contains relevant biological information (see below). Early hybrid experiments combining fMRI with well-established electrophysiological recordings have provided valuable insights into the type of neuronal activity that is driving FH.^13^ However, as electrophysiology lacks cell specificity and interferes with MRI, it is has gradually been replaced by newly developed optical techniques. Light can be guided in a straightforward manner into and out of the magnet, causing no or minimal interference with the fMRI experiment. Further, optical reporter systems such as genetically encoded calcium indicators (GECI) can be rendered cell-specific in a highly selective manner. Thus, optical techniques can facilitate multimodal recordings of cell-specific contributions to neural processing. Their major limitation arises from the interaction of light photons with biological tissue (scattering, attenuation), which limits tissue penetration. Even when using the most favorable wavelength domain, light propagation becomes diffusive at distances exceeding a few hundred micrometers, and spatial information is rapidly lost. Hence, optic recording procedures may need to be invasive, i.e., the brain region of interest may need to be exposed or accessed by inserting an optical fiber. Fiber-based techniques are of particular interest when studying deep brain structure as they significantly reduce procedure invasiveness.

Apart from understanding the nature of the functional readout provided by fMRI and related techniques, studying the interplay of different neuronal structures in controlling information processing across the brain is of key importance in neuroscience. In addition to providing a noninterfering frequency band for complementary imaging, terahertz electromagnetic radiation such as visible light can be employed for stimulation. This methodological variation enhances certainty in the study of brain function compared to sensory stimulation, as predefined signals can be introduced directly at the neuronal level in well-defined brain structures. In particular, this allows the targeted study of neuronal pathways of high biomedical relevance which, however, generate only weak endogenous signal, such as monoaminergic^18^^,^^19^ and neuropeptidergic^20^ pathways. Further, this method of study can complement the computational network modeling of the brain (e.g., Ref. 21) with *in vivo* targeting of node-like systems (see, e.g., Ref. 22) within the spatiotemporal bounds of current methodological feasibility.

As the brain is not endogenously sensitive to light, primary signal transduction needs to be provided via an exogenous manipulation. This is commonly done via expression of light-sensitive bacterial proteins, a process known as optogenetics, and in conjunction with fMRI, as opto-fMRI.^23^ Not surprisingly, the introduction of optogenetics as a tool for highly specific interventions in the behaving animal already constituted a milestone for the study of brain functional architecture.^24^^,^^25^ Moreover, after selective transfection of a specific cell population to express an optogenetic construct, it could be demonstrated that the functional network associated with the target population is discernible in fMRI readouts.^23^^,^^26^

Hence, the combination of fMRI with optical methods is attractive both for linking the response of different CNS cell types to noninvasive imaging readouts and for analyzing the effect of specific cell populations on information processing. In this article, we will first discuss methodological aspects of hybrid fMRI and optical system integration and then focus on these two aspects: multimodal recording of neuronal and astrocytic responses in combination with fMRI (at rest and during sensory stimulation) and imaging of effective connectivity patterns associated with specific neuronal populations. For the latter part, we will particularly focus on serotonergic and dopaminergic signaling elicited by stimulation of the respective deep brain nuclei.

## 2 Methods Overview

### 2.1 Multimodal Measurement

The BOLD fMRI signal is determined by both neurovascular and neurometabolic adaptation, whereas pure hemodynamic readouts such as activity-induced CBF and CBV changes reflect only volume integrated FH responses. Combining the different fMRI readouts with alternative (invasive) measures of stimulus-evoked activity within the neurovascular unit (NVU) provides important insight regarding the interpretation of fMRI signals. Readouts tightly linked to neuronal activity include optical imaging using voltage-sensitive dyes, measurements of calcium (${\mathrm{Ca}}^{2+}$) transients as prompted by membrane depolarization, and assessment of neurotransmitter turnover (glutamate/glutamine). Oxygen and glucose consumption assessment, by contrast, is not neuron-specific, but remains nevertheless of interest with regard to neurometabolic coupling.

Electrophysiological recordings, which provide information on membrane potentials directly and exclusively, have frequently been combined with fMRI. For example, Logothetis et al.^13^ demonstrated a high degree of correlation between the BOLD signal and local field potentials (LFPs) as a measure of overall synaptic input and local processing. While such recordings yield direct insight into the electrical activity of neurons and provide high temporal resolution, they are technologically challenging and do not provide cellular specificity, i.e., other constituents of the NVU cannot be distinguished. An attractive alternative is the measurement of ${\mathrm{Ca}}^{2+}$-transients using optical recordings/imaging in combination with calcium-sensitive dyes^27^ or GECIs,^28^^–^^30^ which allow the assessment of cell-specific contributions to neural processing.

#### 2.1.1 Experimental design: hybrid rationale

The optimal optical method to be combined with the fMRI setup depends on the scientific question to be tackled. Hence, before designing a hybrid setup, the following questions should be addressed:

The first important question in hybrid setup design is: *Does the scientific question require simultaneous data acquisition?* Sequential acquisition with two (or more) modalities may be advantageous if the objects of study are, e.g., evoked brain responses that are highly stereotypic and constant over time. Given a stimulus paradigm that excludes refractory or adaptation effects, one might consider trial-by-trial variability of neural responses to identical stimuli as random noise, meaning that it does not contain relevant biological information. Contributions of random noise can be accounted for by averaging over multiple trials. Under these circumstances, sequential acquisition would be advantageous to avoid trade-offs, which, as discussed below, inherently arise in either modality when using a hybrid measurement setup.

However, recent experimental^31^ and computational studies^32^ suggest that trial-by-trial variability in stimulus-evoked recordings comprises relevant information about the behavioral state and ongoing network dynamics. As such, assuming that activity is largely spontaneous in nature, meaningful correlation of neural and hemodynamic fluctuations would require simultaneous acquisition and thus multimodal readouts of brain activity. This is of course also the case for task-free experiments, in which resting-state networks are determined by analyzing fully endogenous and thus “spontaneous” slow-wave fluctuations.

Further, the majority of the studies mentioned in this review are conducted in rodents, which typically require anesthesia. This affects multimodal experiments twofold: first, the anesthetic modulates spontaneous ${\mathrm{Ca}}^{2+}$ activitiy; and second, the anesthetic changes the breathing pattern and thereby the ${\mathrm{CO}}_{2}$ levels in the bloodstream, which drastically impacts BOLD responses.^33^

The second major consideration is: *Does the scientific question ask for an optimal method providing cellular resolution?* There are two strategies to gather cell-specific information using optical methods: microscopy-based methods, which provide sufficient spatial resolution to identify individual cells; and bulk sampling methods, which permit identification of specific cell population using selective labeling techniques. The fMRI signal of a voxel represents the integrated activity of thousands of neurons. Hence, when investigating the contribution of a specific cell population to the lumped fMRI signal, it may be more appropriate to acquire the bulk signal of a volume comparable to that giving rise to the fMRI signal rather than monitoring the behavior of individual cells within a much smaller FOV. Nevertheless, as already discussed, high spatiotemporal resolution is of relevance for analyzing mechanisms underlying NVC and its dynamics.^14^^–^^16^ For example, MRI/fMRI studies using high spatial resolution ($100\;\mu m^{2}\times 100\;\mu m^{2}$ and $50\;\mu m^{2}\times 50\;\mu m^{2}$, respectively) in anesthetized rats enabled correlating fluctuations of vessel-specific fMRI signals with the intracellular calcium signal measured in neighboring neurons.^18^

Signals reflecting brain activity are typically weak, and the signal-to-noise ratio (SNR) is commonly a limiting factor for true hybrid measurements, i.e., simultaneous recording of signals with two (or more) modalities. Adapting modalities to work in parallel exacts a toll on their performance. Boosting up spatial resolution would further compromise SNR, as the signal is proportional to the volume of the individual voxel sampled. This can be accounted for using high magnetic field strengths and a small MRI receiver coil yielding a high filling factor ($B_{0}=14.1\;T$ and 6-mm diameter radiofrequency surface coil in the study of Ref. 18), which inherently limits the FOV. For most practical cases, SNR constraints favor using the largest voxel (sample) volume possible, and in case spatial resolution is not required, i.e., the bulk optical signal is sufficient to address the scientific question, fiber photometry provides the highest SNR advantage. In fMRI, however, the voxel size cannot be increased arbitrarily, as magnetic field variations produced by different tissue types within a larger voxel (partial volume effects) can degrade the SNR.

A third major question related to hybrid setup design is: *Is data collection from deep-lying brain areas relevant for answering the scientific question at hand?* Deep brain structures are of relevance when studying signal processing in the brain. For example, peripheral sensory input is routed via the spinal cord and subcortical nuclei such as the thalamus to the respective somatosensory cortical area. fMRI, enabling 3D coverage of the whole brain, is ideally suited for such studies. Yet, can we assume that the relationship between cellular activity measures and the hemodynamic fMRI response, which due to accessibility is typically studied in brain cortex, is independent of the brain region studied? This is unlikely, as both the cellular composition of brain tissue as well as the local vascular architecture are region-specific and, hence, the weight of their contributions to the fMRI signal will vary across the brain. In fact, in biophysical modeling of the fMRI signal response, brain region-specific hemodynamic response functions (HRF) have to be considered.^34^ Therefore, we cannot assume that what we have learned for cerebral cortex can be simply extrapolated to other brain regions.

Optical access to deeper brain structures is limited by the nature of light photon interaction with biological tissue (e.g., due to diffusive light propagation and low tissue penetration depth). There are two approaches to study deep-lying structures with optical techniques: the use of diffuse optical tomography (including fluorescence tomography), which reconstructs light source distribution in 3D from projections measured at the tissue surface, but which yields spatial information significantly inferior to MRI.^35^ Alternatively, invasive procedures have to be used, i.e., superficial brain layers covering the region of interest have to be cleared (e.g., via a cortical window^36^^,^^37^ or via the surgical introduction of optical fibers and fiber bundles).^38^ Individual single- or multimode fibers are probably the least invasive of these approaches and may constitute the method of choice for studying subcortical structures including basal ganglia and brainstem nuclei.

#### 2.1.2 Experimental design: hybrid variants

**Fig. 1:**
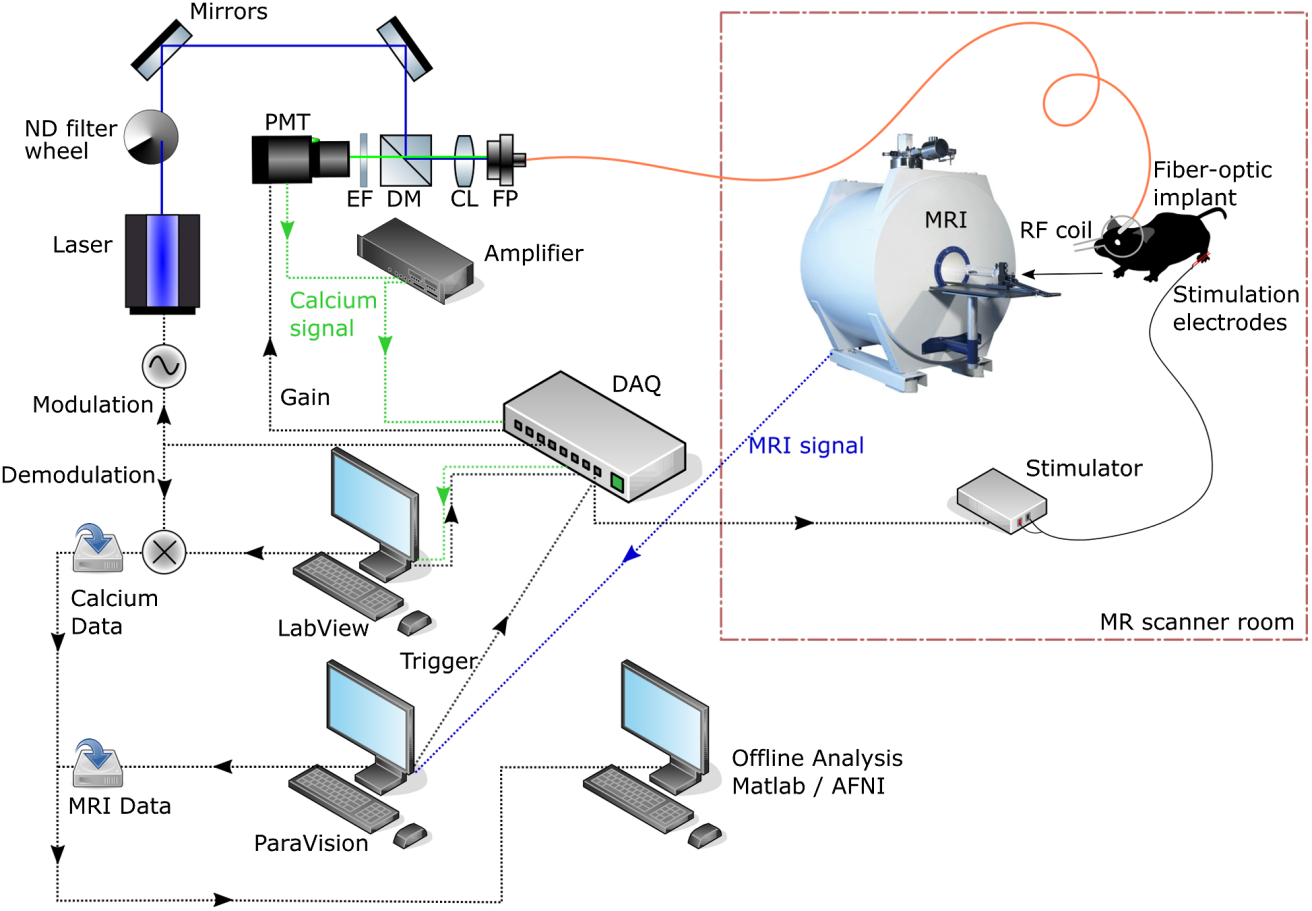
Fiber photometry is based on an advanced yet robust multicomponent system leveraging optical fiber light transmission to separate MR and optical instrumentation. A laser beam is coupled into a fiber-optic patch cable that is connected to an implant on the mouse; fluorescent emissions are guided back through the same fiber; the entire optical setup is located outside of the MR scanner room. MRI volumes, the fluorescence time course, and the stimulation protocol are then combined in offline analysis. Abbreviations: PMT, photomultiplier tube; EF, emission filter; DM, dichroic mirror; CL, coupling lens; FP, fiber port; ND, neutral density; DAQ, data acquisition device. Adapted from Ref. 39.

To provide adequate solutions for multiple of the use case variants delineated above, multiple variations of integrating light modalities with fMRI have been developed, which include the following:

Fiber photometry (Fig. 1) is ideally suited for simultaneous readout with fMRI and has so far been the most popular technique for *in-vivo* hybrid recordings in rodents (see Table 1). An optical fiber can guide the laser beam into the MR bore, and the fluorescence signals to a detector outside of the magnetic field, so the two methods do not interfere with each other. While a single multimode fiber lacks spatial resolution, the placement of the fiber tip allows regional selectivity for a given brain region. Further spatial specificity can be gained by selecting the core diameter and numerical aperture of the multimode fiber to adjust the size of the cone where light is emitted and collected, such that it matches the area of interest. In addition, an array of fibers can be implanted so as to target multiple brain regions of interest, including subcortical areas.^48^ Cell type or cell compartment specificity can be attained by expressing GECIs under the appropriate promoters.^49^^,^^50^ The hardware for fiber photometry is largely interchangeable with the optogenetic stimulation tools discussed in the subsequent sections. Thus, the two methods can readily be combined, allowing multimodal readouts under optogenetic control of defined neuronal circuits.^43^^,^^51^

**Table 1:** Studies using hybrid systems for optical ${\mathrm{Ca}}^{2+}$-recording and fMRI *in vivo*.

Author, year	Optical method	fMRI method	Target cells	Brain region	Paradigm	Anesthesia	Species
Schulz et al. 2012^40^	FP	BOLD	Unspecific ${\mathrm{Ca}}^{2+}$	fp./hp. S1	fp./hp. stim.	iso.	Rat
Liang et al. 2017^41^	FP	BOLD	Neurons	SC	Visual stim.	med.	Rat
Schwalm et al. 2017^42^	FP	BOLD	Neurons	fp. S1, Po	RS	iso.	Rat
He et al. 2018^18^	FP	BOLD, CBV (s.v.)	Neurons	fp. S1, vibrissa S1	fp., RS	α-chloralose	Rat
Schlegel et al. 2018^39^	FP	BOLD	Neurons, astrocytes	hp. S1	hp. stim., RS	iso.	Mouse
Wang et al. 2018^43^	FP	BOLD	Astrocytes	fp. S1	RS	ur.	Rat
	FP	BOLD	Astrocytes	fp. S1	fp. stim.	med.	Rat
	FP	BOLD	Neurons, astrocytes	fp. S1	fp. stim., RS	α-chloralose	Rat
Chen et al. 2019^44^	FP	BOLD	Neurons	Barrel S1	Optogenetic stim.	α-chloralose	Rat
Tong et al. 2019^45^	FP^a^	BOLD	Neurons	SC, LGN	Visual stim., RS	med.	Rat
Van Alst et al. 2019^33^	FP	BOLD, ASL	Neurons	fp. S1	fp. stim.	iso.-med.	Rat
Lake et al. 2020^46^	WI	BOLD	Excitatory neurons	Whole cortex	hp. stim.	iso.	Mouse
Pais-Roldan et al. 2020^47^	FP	BOLD	Neurons	Cingulate cortex	Pupil size correlated RS	α-chloralose	Rat

Abbreviations: ASL, arterial spin labeling; fp., forepaw; FP, fiber photometry; hp., hindpaw; iso., isoflurane; LGN, lateral geniculate nucleus; med., medetomidine; Po, posterior thalamic nucleus; RS, resting state; S1, primary somateosensory cortex; SC, superior colliculus; stim., stimulation; s.v., single-vessel; ur., urethane; WI, widefield imaging.

a Within-subject two-site optical measurement.

Optical microscopy used in conjunction with fMRI combines the excellent spatiotemporal resolution of fluorescence microscopy with the full brain coverage of fMRI and thus provides a useful bridge between the microscopic, mesoscopic, and macroscopic scale. However, optical imaging systems contain many sensitive electronic components and actuators that cannot operate in an MR environment. Designing hybrid systems in which the optical equipment satisfies the material and spatial constraints of MRI, thus remains an ongoing challenge. In particular, canonical RF coil designs may also interfere with the light path. Nevertheless, a proof-of-concept for an MR-compatible two-photon microscope has been developed, in which the optical components are connected to the MR bore via a fiber light guide.^52^ In a recent study, a fiber-optic bundle was used to project the fluorescence signals on a camera outside the MR room, allowing widefield mesoscopic imaging of the entire mouse cortex.^46^ Another prototype used a miniaturized MR-compatible camera, allowing a one-photon microscope to operate within the MR bore.^53^

Diffuse optical imaging combined with fMRI presents an alternative to fluorescence-based recordings, as the absorption properties of hemoglobin can be used as a functional readout of blood volume and oxygenation changes. NIRS, owing to the attractive properties of near-infrared light, has found widespread use as a noninvasive tool in clinical applications.^54^ The high penetration depth, which enables it to pass through even the human skull, and the ability to provide absolute quantification of hemodynamic parameters, have made it an early tool of choice to validate assumptions made with BOLD fMRI.^55^ Hybrid NIRS/fMRI systems can also expand the scope of MRI applications without the need for exogenous contrast agents.^56^ Typically, a setup consists of one or more light source/photodetector pairs, which, as in fiber-photometry, can be connected via fiber-optic cables. Typically, the recordings are not spatially resolved, as diffusive light paths inherently limit the resolution, though camera-based systems have been developed.^57^ Ultimately, as another hemodynamic readout, NIRS cannot provide the complementary information needed to separate the various contributions to BOLD fMRI, which thus limits its utility for NVC research.

### 2.2 Optogenetic Stimulation

Discerning how specific cell types affect brain wide functional responses is essential for elucidating the interconnection of brain networks and their roles in information processing and relay. As already described, light is ideally suited for this purpose due to its minimal interference with the imaging readout method (see Sec. 1) and the fact that brain tissue is not intrinsically sensitive to light. Manifold molecular biological techniques, rendering tissue specifically susceptible to light photons after light-sensitive sensor protein introduction via genetic engineering, are therefore fully accessible for fMRI. The combination of optogenetics and fMRI has thus become a method of choice across neuoscientific applications (see Table 2), general concepts and trends of which we further lay out.

**Table 2:** Literature overview of opto-fMRI experiments. All optogenetic constructs are excitatory and have rapid kinetics, unless otherwise noted. Cre-LoxP selection for expression is prepended in parentheses to the promoter, when applicable. If the experiment uses vector injection as opposed to constitutive optogenetic construct expression and the injection site differs from the stimulation site, the injection site is prepended in parentheses. Paradigms detail in parentheses the characteristics of the smallest structured stimulation train, and original sources should be consulted, as ON periods may contain additional pauses between stimulation trains. Step function opsins do not possess canonical within-stimulation-period parameters.

Author, year	Optogenetic construct	fMRI method	Promoters	Brain region	Paradigm ($\tau_{p}$, $f_{\mathrm{rep}}$)	Anesthesia	Species
Lee et al. 2010^23^	ChR2(H134R)	BOLD	CaMKIIα	Th.	Block (15 ms, 20 hz)	iso.-$N_{2}O$	Rat
	ChR2(H134R)	BOLD	CaMKIIα	M1	Block (15 ms, 20 hz)	iso.-$N_{2}O$	Rat
	ChR2(H134R)	BOLD	CaMKIIα	(M1)Th.	Block (15 ms, 20 hz)	iso.-$N_{2}O$	Rat
	ChR2(H134R)	BOLD	(PV-Cre)CaMKIIα	M1	Block (15 ms, 20 hz)	iso.-$N_{2}O$	Rat
Desai et al. 2011^26^	ChR2	BOLD	CaMKIIα	S1	Block (8 ms, 40 hz)	iso., awake	Rat
	ChR2	BOLD	Thy1	S1	Block (8 ms, 40 hz)	iso., awake	Rat
Gerits et al. 2012^58^	ChR2	CBV	CaMKIIα	F5	Block (8 ms, 40 hz)	Awake	Rhesus m.
	ChR2	CBV	CaMKIIα	FEF	Block (8 ms, 40 hz)	Awake	Rhesus m.
Kahn et al. 2013^59^	ChR2	BOLD	Thy-1	S1	Block (2.7 ms, 8 to 80 hz)	iso.	Mouse
Weitz et al. 2015^60^	ChR2(H134R)	BOLD	CaMKIIa	DH	Block (5 to –50 ms, 6 to 60 hz)	iso.-$N_{2}O$	Rat
	ChR2(H134R)	BOLD	CaMKIIa	IH	Block (5 to 50 ms, 6 to 60 hz)	iso.-$N_{2}O$	Rat
Iordanova et al. 2015^61^	ChR2(H134R)	BOLD	CaMKIIα	S1	Block (5 to 50 ms, 3 to 20 hz)	iso.	Rat
Liang et al. 2015^62^	ChR2(H134R)	BOLD	CaMKIIα	IL mPFC	Block, ER (10 hz to 20 ms, 10 hz)	iso., awake	Rat
Duffy et al. 2015^63^	ChR2(H134R)	BOLD	CaMKIIα	IH	Block (15 ms, 20 hz)	med.	Rat
Ferenczi et al. 2016^64^	ChR2(H134R)	BOLD	(Th-Cre)EF1α	VTA	Block (10 ms, 20 hz)	Awake	Rat
	eNpHR3.0^a^	BOLD	(Th-Cre)EF1α	VTA	Block (10 ms, 20 hz)	Awake	Rat
	${C1V1}_{TT}$	BOLD	(Th-Cre)EF1α	VTA	Block (10 ms, 20 hz)	Awake	Rat
	SSFO^b^	BOLD	(Th-Cre)EF1α	PFC	Block	Awake	Rat
Lohani et al. 2017^19^	ChR2	BOLD, CBV	(Th-Cre)EF1α	VTA	Block (5 ms, 20 hz)	iso.	Rat
Aksenov et al. 2016^65^	hChR2(H134R)	BOLD	CaMKIIα	barrel S1	Block (unreported, 50 hz)	Awake	Rabbit
Albaugh et al. 2016^66^	hChR2(H134R)	CBV	CaMKIIα	NAcc	Block (10 ms, 40 hz)	iso.-med.	Rat
Christie et al. 2017^67^	hChR2(H134R)	BOLD	CamKIIα	hp. S1	ER (10 ms, N/A)	α-chloralose	Rat
Takata et al. 2018^68^	ChR2(C128S)^b^	BOLD	Chrm4	V1 neurons	Block	Awake	Mouse
	ChR2(C128S)^b^	BOLD	Mlc1	V1 astrocytes	Block	Awake	Mouse
Choe et al. 2018^69^	Archaerhodopsin^a^	BOLD	(L7-Cre)CAG	fp. cerebellum	Block (5 to 20 hz)	med.	Mouse
Brocka et al. 2018^70^	C1V1(E162T)	BOLD	CamKIIα	VTA	Block (10 ms, 25 hz)	med.	Rat
	hChR2(H134R)	BOLD	(Th-Cre)EF1α	VTA	Block (10 ms, 25 hz)	med.	Rat
Grandjean et al. 2019^22^	hChR2(H134R)	BOLD, CBV	(ePet-Cre)EF1α	DR	Block (5 ms, 20 hz)	iso.-med.	Mouse
Leong et al. 2019^71^	ChR2(H134R)	BOLD	CaMKIIα	MVN	Block (10 ms, 20 hz)	iso.	Rat
Y. Chen et al. 2019^44^	ChR2	CBV	CAG	Th.	Block (5 to 20 ms, 3 to 10 hz)	α-chloralose	Rat
Albers et al. 2019^72^	C1V1(E122T/E112T)	BOLD, dfMRI	CamKIIα	fp. S1	Block (10 ms, 9 hz)	med.	Rat
X. Chen et al. 2019^73^	hChR2(H134R)	BOLD, CBV (s.v.)	CAG	CA1	Block, ER (1 to 20 ms, 1 to 50 hz)	α-chloralose	Rat
	${C1V1}_{TT}$	BOLD, CBV (s.v.)	CaMKIIα	CA1	Block, ER (1 to 20 ms, 1 to 50 hz)	α-chloralose	Rat
Y. Chen et al. 2020^74^	hChR2(H134R)	BOLD	CaMKII	(barrel S1)CC	Block (1 h to 40 hz, 1 to 20 ms)	α-chloralose	Rat
Ioanas et al. 2022^75^	hChR2(H134R)	CBV	(DAT-Cre)EF1α	VTA	ER, block (5 ms 15 to 25 hz)	iso.-med.	Mouse
Ioanas et al. 2021^76^	hChR2(H134R)	CBV	(ePet-Cre)EF1α	DR	Block (5 ms, 20 hz)	iso.-med.	Mouse
Cover et al., 2021^77^	ChR2(H143R)	BOLD	hSyn	IL mPFC	ER (5 ms, 25 hz)	Awake	Mouse

Abbreviations: C1V1, *Chlamydomonas reinhardtii* and *Volvox carteri* chimeric channelrhodopsin, with E122T and E162T mutations; CA1, hippocampus cornu ammonis 1; CAG, cytalomegavirus early enhancer/chicken β-actin; CaMKII, calcium-calmodulin kinase IIα; CC, corpus callosum; CHRM4, cholinergic receptor suscarinic 4; dfMRI, diffusion fMRI; DH, dorsal hippocampus; DR, dorsal raphe; EF1α, human elongation factor 1 alpha; eNpHR3.0, *Chlamydomonas reinhardtii* halorhodopsin variant; ePET, Pet-1 enhancer; ER, event-related; FEF, frontal eyefield; $f_{\mathrm{rep}}$, laser pulse repetition frequency; hChR2, humanized Channelrhodopsin 2, variant in parentheses; hSyn, human synapsin 1 promoter; IH, intermediate hippocampus; IL mPFC, infralimbic medial prefrontal cortex; LH, lateral hypothalamus; Mlc1, myosin alkali light chains; MVN, medial vestibular nucleus; NAcc, nucleus accumbens; PFC, prefrontal cortex; PV, parvalbumin promoter; rhesus m., rhesus macaque; SSFO, stabilized step function opsin; s.v., single-vessel; TH, tyrosine hydroxylase; Thy1, thymus cell antigen 1; Th., thalamus; $\tau_{p}$., laser pulse width; ur., urethane.

a Inhibitory.

b Step-function opsin, excitatory.

#### 2.2.1 Vectors

Available optogenetic signal transducers include ion channels, such as channelrhodopsins,^78^ and ion pumps, such as halorhodopsins.^79^ Both stimulation and inhibition can be elicited via such constructs, the former most commonly via cation-permeable channelrhodopsins (e.g., ChR2^80^) and the latter via anion-transporting halorhodopsins (e.g., eNpHR3^81^) or anion-permeable channelrhodopsins (e.g., GtACRs^82^). A further methodological refinement opportunity is provided by step-function opsins, which encompass both anion^83^ and cation^84^ channels. These constructs remain in an altered conformational state (minute-range off-kinetic time constants) following light stimulation and can be returned to the “inactive” conformational state via light stimulation at a different wavelength—as opposed to classic channelrhodopsins, which effectively convert into the “active” state only during light input (millisecond-range off-kinetic time constants).^85^

**Fig. 2:**
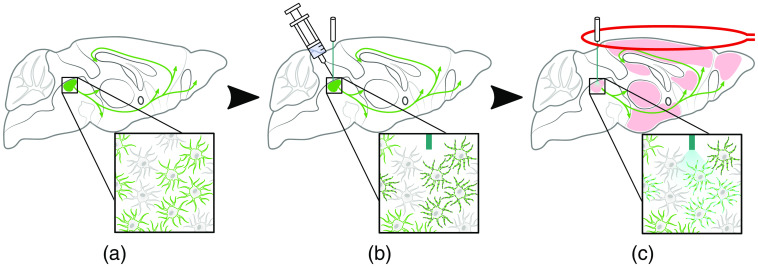
Graphical representation of a single-session opto-fMRI workflow, highlighting its sequential integration of optogenetics and fMRI. Panels show (a) the usage of a transgenic strain expressing Cre recombinase, (b) viral vector delivery of an optogenetic construct using the Cre/LoxP system and optical cannula implantation targeting the entire transfected system, and (c) fMRI measurement with concurrent light stimulation. Green represents cells with Cre expression (green arrows indicate structural projections), dark gray dots represent optogenetic construct expression, cyan represents light stimulation and light-evoked postsynaptic activity at the stimulation site, and pink represents MR signal. Figure adapted from Ref. 76, with permission.

Opto-fMRI applications consist of the sequential integration of optogenetic targeting and the delivery of light concomitant with fMRI measurement. Though specific targeting for both vector delivery and stimulation can vary greatly, a representative use case of prevalent practices is presented graphically in Fig. 2. In this example, the colocalization of cell bodies of a widely projecting neurotransmitter system in a brainstem nucleus is leveraged to stimulate a wide efferent spectrum with a coherent signal.

Vector delivery methods for opto-fMRI use either the Cre-LoxP system in conjunction with AAV (adeno-associated virus) vectors or lentiviral vectors^58^—whereby the former provides the advantage of decoupling the cell-type selection from the optogenetic construct, allowing greater interrogation flexibility without the need for custom vector production. While Cre-LoxP-based targeting commonly employs transgenic lines, the Cre construct can itself be delivered via a viral vector—though available virus preparation libraries are more constrained, and there are additional restrictions on promoter length, particularly if delivered via AAV.

A critical aspect for the success of opto-fMRI experiments is the elicited signal amplitude, as the intrinsically low SNR of the method^86^ constitues a major challenge. High expression levels, sufficient photon flux density at the target site, and a stimulation protocol yielding high contrast are essential for obtaining reliable signals.

#### 2.2.2 Experimental design: setup

The foremost consideration in applying opto-fMRI is selecting the correct biological features for both stimulation and imaging. While the breadth of neurosicentific applications cannot be sufficiently detailed without a review of all neurobiology, a series of guidelines can be formulated based on the characteristics of the method. Conceptually, opto-fMRI uses targeted, invasive, and high-temporal-resolution stimulation to drive a set of neurons, combined with a whole-brain, noninvasive, medium-temporal-resolution read-out method. Thus, an important question is whether targeted stimulation is relevant with respect to whole brain imaging. As a consequence, the ideal setting for leveraging the full potential of the technology is targeting widely projecting neurotransmitter systems to investigate their effects on neuronal activity at the whole-brain level. This is substantiated by extant opto-fMRI literature, in which the stimulation of wide efferent spectrum structures such as monoaminergic systems^19^^,^^22^^,^^75^^,^^76^ and long-range glutamatergic projections^62^^,^^87^ are prominently paired with whole-brain imaging and analysis. A selected structure with a wide efferent spectrum may however only evoke local activity, such as has been the case for the nucleus accumbens,^66^ which provides a setting for contrasting structural, electrically evoked, and optogenetic excitation kinetics. Opto-fMRI has, on the other hand, also been used with the express intent of stimulating and measuring localized activity, such as in primarily self-projecting cortical regions or in the hippocampus, which is prone to local seizure effects.^63^^,^^88^^,^^89^ Such applications, although not leveraging the full spatial potential of fMRI, provide relevant methodological information, support in technology development,^63^ and yield relevant information with regard to the nature of the fMRI signal.^23^^,^^59^^,^^73^

While fMRI can be freely combined with any optogenetic technologies, the exigences imposed by MRI scanner access specifically encourage the use of the most established and extensively characterized constructs. As such, constructs of great clinical interest, such as anion-permeable channelrhodopsins and step-function opsins, have respectively seen no or little^64^^,^^68^ opto-fMRI use and present promising opportunities for future research.

A key constraint to leveraging the full versatility of optogenetics, and thus of opto-fMRI, is the application of the technology in species with low or no availability of transgenic lines. The highly flexible Cre-LoxP/AAV vector delivery method, which allows the decoupling of signal transducer variants from cell-type selection, is contingent on transgenic lines expressing Cre recombinase under the desired cell-type-specific promoter. Large libraries for such lines are available for the mouse, and increasingly, but to a lesser extent, for the rat. Other model animals, and particularly higher primates, do not offer any comparable level of access, and consequently, opto-fMRI application in these settings presupposes the use of more unwieldy custom lentiviral vectors, as well as close consideration of the trade-off between promoter length and expression characteristics.^58^^,^^90^

#### 2.2.3 Experimental design: stimulation protocols

Optogenetics offers considerable flexibility with regard to the stimulation protocol, apt usage of which can considerably enhance the SNR for opto-fMRI. A fundamental distinction is made in the field of fMRI between “event-related” and “block” designs, which refer to the distribution of stimulation events over the duration of the experiment. Nominally, a design is deemed event-related if the stimulation (ON) period consists of a single event or is otherwise equal to or shorter than the acquisition TR—with longer ON durations being deemed block designs. Event-related designs lend themselves to sequence randomization, as they provide low but similar contrast upon variation, more closely resembling physiological activation. While such protocols can be employed in opto-fMRI (and may consist of single events^67^ or short trains^62^) block designs are generally preferred due to the ability to deliver superior contrast.^22^^,^^75^^,^^91^^,^^92^ In self-stimulation applications, short events can be concentrated into self-stimulation ON periods, yielding designs which are nominally event-related but may residually benefit from block-design contrast characteristics depending on the statistical analysis.^77^

The precise time-sequence of a block design can further be optimized with respect to both its contrast characteristics and its feasibility for the given biological system being targeted. The contrast characteristics of the stimulation protocol refer to the theoretical quality of its statistical estimation in the general linear model (GLM), which is the analysis method most commonly used to resolve spatial response patterns based on a stimulation time course. Experimental parameters that bear on the fitness of a stimulation time course include experiment length, temporal frequency band reliability (lower frequency bands in particular may be considered unreliable in fMRI due to drift), as well as the impulse response function, which differs greatly with respect to contrasts (BOLD, CBV, or custom functional contrast agents). These parameters can be submitted to genetic-algorithm optimization workows,^93^^,^^94^ which can produce highly performing stimulation time courses.

The internal structure of stimulus blocks can also be adapted to increase the contrast generated in fMRI, though this may produce varying degrees of physiological comparability in the elicited activity mode. The intrinsic SNR constraints of fMRI, particularly when performed without cryogenic coils, however, prompt stimulus train optimization toward evoking the maximal activity level compatible with tissue preservation. Stimulation protocols for opto-fMRI, based predominantly on in-house empirical optimization, generally span 10 to 100 Hz in frequency and 2 to 20 ms in pulse width.^19^^,^^22^^,^^62^^,^^66^^,^^95^ Such optimizations are formalized in a number of ancillary methodological investigations, which predominantly identify strong effects for stimulation frequency and weak effects for pulse width variations.^22^^,^^44^^,^^73^^,^^95^ While this is consistent with theoretical considerations arising from extant construct off-kinetics^85^—and thus constitutes the best working hypothesis for stimulus structure optimization—some studies do not reproduce this trend,^61^^,^^74^ and further results indicate nonlinear and differential neuronal population recruitment based on pulse frequency.^60^ Far from simply being a technical parameter for which basic optimization heuristics could reliably be translated into *a priori* optimized protocols across brain areas, stimulus structure may also be leveraged as a flexible tool for the exploration of differential signal propagation.

A ceiling for the maximization of optogenetically evoked signal can be estimated with regard to heating artefacts, which can arise following light stimulation.^96^ Heating artifacts are of particular concern for ion-pump optogenetic constructs, as these require considerably stronger light stimulation.^97^ Systematic study of the phenomenon initially reported its emergence at approximately $20\;\mathrm{mW}\;{\mathrm{mm}}^{-2}$ average light deposition per second (445- and 532-nm light stimulation),^89^ and subsequently at as little as $9\;\mathrm{mW}\;{\mathrm{mm}}^{-2}$ (445-nm light stimulation).^67^ Later still, initial trends were corroborated by a systematic study estimating the emergence of heating artifacts bewteen 19.5 and $25\;\mathrm{mW}\;{\mathrm{mm}}^{-2}$ (552-nm light stimulation).^72^ These estimates may, however, vary at different wavelengths, across brain areas, and be contingent on within-pulse laser power (Cf. Refs. 72 and 67) and stimulation block duration (in addition to the time-averaged light deposition)—and may further depend on the optic cannula diameter in a nonlinear fashion.^98^ Heating artifacts (alongside unspecific visual-activation-based signal) thus remain a significant methodological risk in opto-fMRI. In addition to preliminary empirical verification in a control group, these confounds can be mitigated in GLM analysis given sufficient control group size.^75^^,^^91^^,^^92^

An important consideration throughout stimulation protocols is that optogenetics can drive, but cannot clamp activity. This means that while both excitation and inhibition can be elicited, this does not happen in a complementary manner. In the absence of activation-inducing stimuli, cells are not inactivated, but rebound toward endogenous network activity. Similarly, in the absence of inactivation-inducing stimuli, cells will also rebound toward normal endogenous network activity. This constraint even applies to step-function opsins,^84^ which work as ion channels, and whose primary advantage is requiring minimal light to switch into an “open channel” state. Step-function optogenetic techonlogy is theoretically feasible for providing clamping functionality with minimal light deposition in a fused-protein assay, though such tools are not currently available, and functionality approaching clamping is only available via construct codelivery.^99^

### 2.3 Implant Design

A common though easily overlooked constraint in small animal applications, which affects both multimodal recording and optogenetic stimulation experiments, is the potential for setup incompatibilities between optical tools and the cutting edge of fMRI technologies. This manifests itself in implant/coil incompatibility, whereby surface coils—cryogenic coils in particular—constrain the positioning of animals with optical implants as are canonically used for light-based measurement or stimulation. Manufacturing as well as operation protocol developments permit pitch and yaw variable targeting of structures,^100^ though large-scale hybrid measurement or opto-fMRI studies leveraging cryogenic surface coil capabilities have not yet been published. An additional unexpected pitfall is the magnetic susceptibility of dental cement, which is commonly used to stabilize optic cannulae or skull windows, particularly with regard to longitudinal applications. For clinical practice, dental cements are commonly adulterated with metal oxides and silicates to produce radioopaque characteristics,^101^ which can also lead to artifacts in MRI.^102^ This issue can generally be avoided using dental cements not explicitly labeled as radioopaque, but as radioopacity is a desired diagnostic trait, pre-implantation cement testing is recommended. Further, during any implantation procedure, additional care must be taken to not enclose any air pockets or blood clots, as those might similarly introduce susceptibility artifacts in MRI.

## 3 Multimodal Readouts of Brain Activity: Insights into Neurovascular Coupling

### 3.1 Components of Neurovascular Coupling

**Fig. 3:**
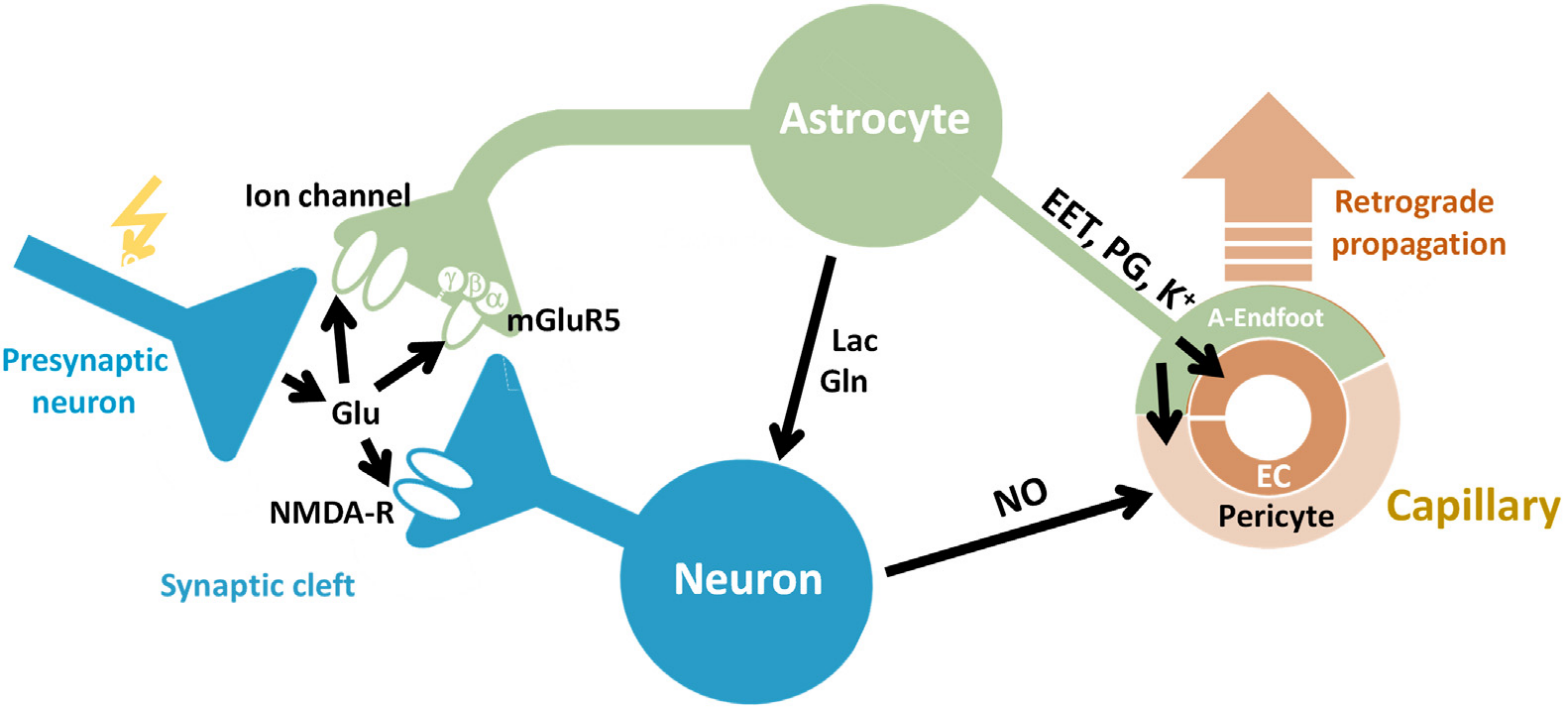
Schematic of cellular interactions mediating neurovascular coupling. Excitatory input triggers synaptic release of glutamate (Glu), which activates neuronal NMDA-R as well as astrocytic ion channels and metabotropic Glu receptors (e.g., mGluR5), prompting the release of vasodilator substances such as NO, EETs, PGs. The vasoactive compounds interact with capillary pericytes (and arteriole and pial artery smooth muscle cells). Local capillary dilation may also result from direct interaction with EC and then be backpropagated to feeding arteries/arterioles via hyperpolarization and mediators such as NO.

The basic NVU consists of neuron, astrocyte, and vascular cells [such as endothelial cells (EC), pericytes, and smooth muscle cells] and is schematically summarized in Fig. 3. The concept of CBF regulation in response to an increased energy demand by active neurons has been revisited in view of the fact that neurons can maintain their initial activity without requiring additional blood supply and, as a consequence, CBF responses are slow. Neurotransmitter-mediated signaling plays a major role in regulating CBF, the main excitatory neurotransmitter being glutamate. It has been suggested that increased extracellular levels of glutamate following its release at synapses switch astrocytes to anaerobic glycolysis. The relative amount of $O_{2}$ available for neurons thereby increases, while astrocytic lactate would serve as an energy substrate for neurons (a process known as the “astrocyte-neuron lactate shuttle”).^103^ Demonstration of a lactate gradient from astrocytes to neurons supports this hypothesis.^104^ CBF adaptation would then serve to replenish the (astrocytic) energy reservoirs, but also to maintain function during prolonged stimulation.^40^^,^^105^

#### 3.1.1 Astrocytes as mediators of functional hyperemia

The involvement of astrocytes in NVC has long eluded electrophysiological recordings, as they are electrically inexcitable. However, given their key location within the NVU, engulfing the synapses and covering the vasculature with their end-feet, it appears plausible that astrocytes are involved in FH to an extent beyond the mere role of an energy reservoir. Glutamate mediated signaling leads to activation of neuronal N-methyl-D-aspartate receptors (NMDA-R) as well as astrocytic metabotropic glutamate receptors (mGluR) and ion channels.^106^ Downstream processes involve the activation of neuronal nitric oxide synthase and astrocytic phospholipase A2 (PLA2), leading to the formation of nitric oxide (NO) and arachidonic acid derivatives (prostaglandins [PGs] and epoxyeicosatrienoic acids [EETs])—compounds that are known vasodilators.^106^ These early findings provided a strong indication of astrocyte involvement in FH.

**Fig. 4:**
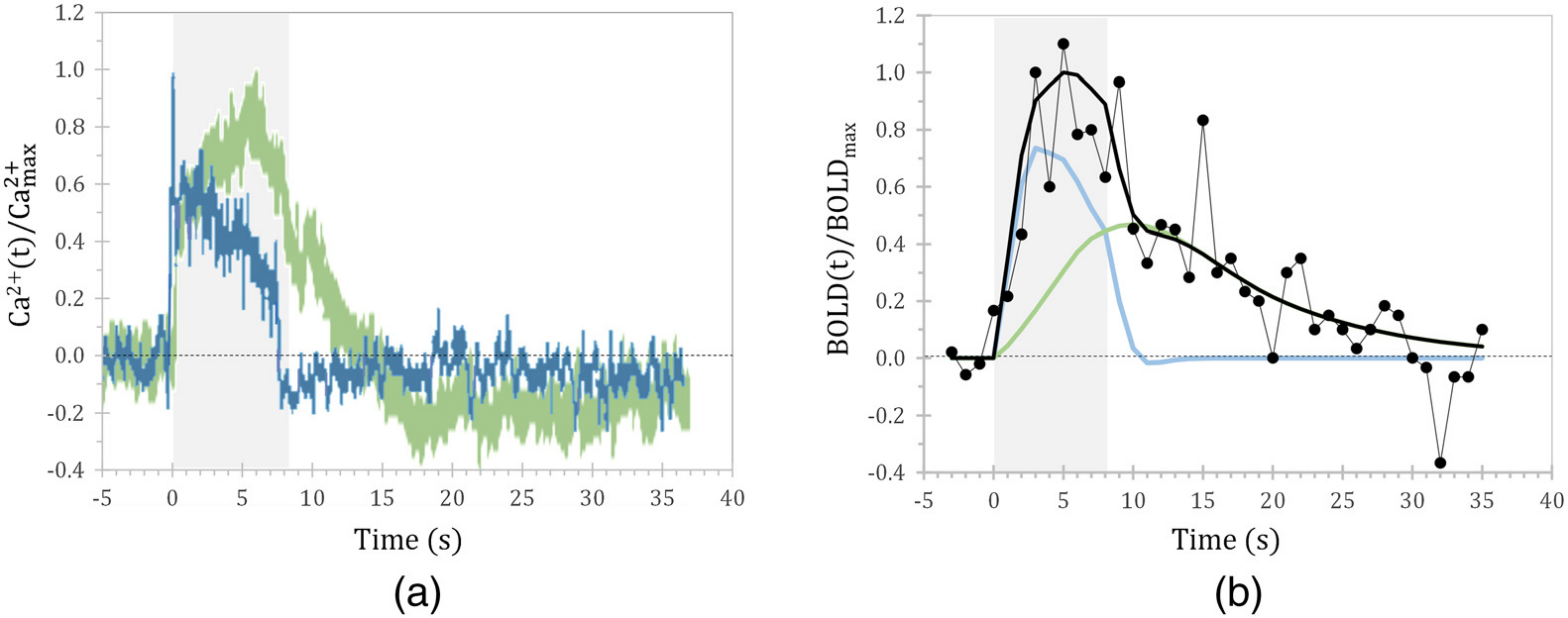
Neuronal and astrocytic signals differ in time course, adding up to the cumulative BOLD response. Forepaw stimulation a mouse under ketamine/xylazine anesthesia, stimulated in 8 s blocks (gray shaded area), with an internal frequency of 3 Hz, an amplitude of 0.7 mA, and a pulse duration of 0.5 ms. (a) Normalized ${\mathrm{Ca}}^{2+}$ transients of neuron (blue) and astrocyte (green) population. Note poststimulus undershoot in astrocytic ${\mathrm{Ca}}^{2+}$ response. (b) BOLD response with black dots indicating experimental data points and thick black solid fitted curve comprising weighted contributions from neurons (blue) and astrocytes (green) contribution as derived from ${\mathrm{Ca}}^{2+}$ recordings convolved with the respective HRF. The HRFs were assumed as cell-type specific gamma-variate functions. Adapted from Skachokova et al. 2021 (pending publication), with permission.

Investigating the intricate role of astrocytes in the generation of BOLD signals is a prime example of a research question that strongly benefits from hybrid optical/MRI approaches. Their involvement could be demonstrated by combining neuronal and astrocytic ${\mathrm{Ca}}^{2+}$ recordings with BOLD fMRI.^40^ Previous studies have shown a sluggish astrocytic ${\mathrm{Ca}}^{2+}$ response to somatosensory stimuli, with a much longer time-to-peak and decay time relative to the neuronal response [Fig. 4(a)]. Such fiber-optic bulk recordings represent the averaged activity of all astrocytes in the area around the fiber tip. It should be noted that individual astrocytes can vary in their temporal characteristics, which can be observed using two-photon microscopy.^107^ Yet, within the spatial scale of BOLD fMRI voxels, it becomes apparent that the kinetics of the summed astrocytic ${\mathrm{Ca}}^{2+}$ response correspond to the later phases of the BOLD response [Fig. 4(b)]—i.e., the prolonged signal elevation after stimulus cessation, and the slow return to baseline. These so-called nonlinear components of the BOLD response have long remained enigmatic as they do not linearly scale with the neuronal response, unlike the BOLD amplitude and time to peak. A growing body of NVC research aims to characterize the diverse roles performed by astrocytes. Recordings of intrinsic astrocytic ${\mathrm{Ca}}^{2+}$ activity concurrent with BOLD fMRI^43^ have found spontaneous ${\mathrm{Ca}}^{2+}$ transients to be coupled with cortex-wide negative BOLD signals and a positive BOLD signal in the thalamus. Subsequent elctrophysiology confirmed that thalamic activity preceded the ${\mathrm{Ca}}^{2+}$ transients, which hints at astrocytes acting as mediators of thalamic regulation of cortical states. Further, optogenetic stimulation of astrocytes has found to be sufficient to evoke positive BOLD responses, without significant activation of nearby neurons.^68^ While the physiological role of such independent astrocytic activation is still unclear, it may provide an explanation for certain task-related hemodynamic responses that occur without neuronal activity.^108^

#### 3.1.2 Vasodilatory signals backpropagate along the vasculature

Electrical forepaw stimulation was shown to prompt a local dilation of the capillary bed and an increase in the velocity of red blood cells indicative of decreased vascular resistance.^109^ Whether this dilation is passive or active is currently unclear, though the effect appears to be controlled by pericytes.^110^^,^^111^ Recent data support the important role of pericytes in NVC, demonstrating that astrocytes signal to pericytes rather than arterioles,^106^ and that pericyte degeneration leads to neurovascular uncoupling.^112^

While neuronal and astrocytic vasodilatory signaling is focused to the site of activity, spatiotemporal analysis of vascular responses revealed involvement of vessels at distances larger than 1 mm^17^^,^^113^ via retrograde propagation of vasodilation.^114^ The EC layer constitutes the obvious guidance structure for signal backpropagation to feeding arterioles and pial arteries, as disruption of EC signaling led to significantly reduced FH. Two vasodilatory mechanisms have been suggested: a fast process mediated by endothelial hyperpolarization and a slow process associated with the release of vasodilator compounds.^17^

Taken together, FH arises as combination of different mechanisms affecting vascular segments in a differential manner.^17^^,^^105^^,^^106^ Neurotransmitter (glutamate) release triggers a sequence of events that lead to an orchestrated response of neurons, astrocytes, and vascular cells (ECs, pericytes, and smooth muscle cells) causing local FH. The vasodilatory stimulus is then backpropagated to arterioles and pial arteries via EC signaling. As the vasculature is dilating along the entire path of this backpropagation, the resulting spatial blurring can make fMRI responses appear more widespread than the underlying neural activitiy.^115^ In addition, the vascular organization differs greatly across brain regions, which may be a key contributor to the regional variability of the HRF observed in fMRI studies.^34^ To date, studies involving vascular signal propagation have been exclusively conducted with high-resolution microscopy, focusing on individual blood vessels. With the continual improvements of GECIs and gene targeting, it has become possible to apply the multimodal techniques discussed in this review to other cell types, such as various vascular cells.^116^ Given the integrative nature of the fMRI signal, multimodal recording of activity-induced cellular responses becomes mandatory for the deconvolution of individual contributions governing the fMRI signal response.

#### 3.1.3 Multimodal imaging for the refinement of forward modeling

**Fig. 5:**
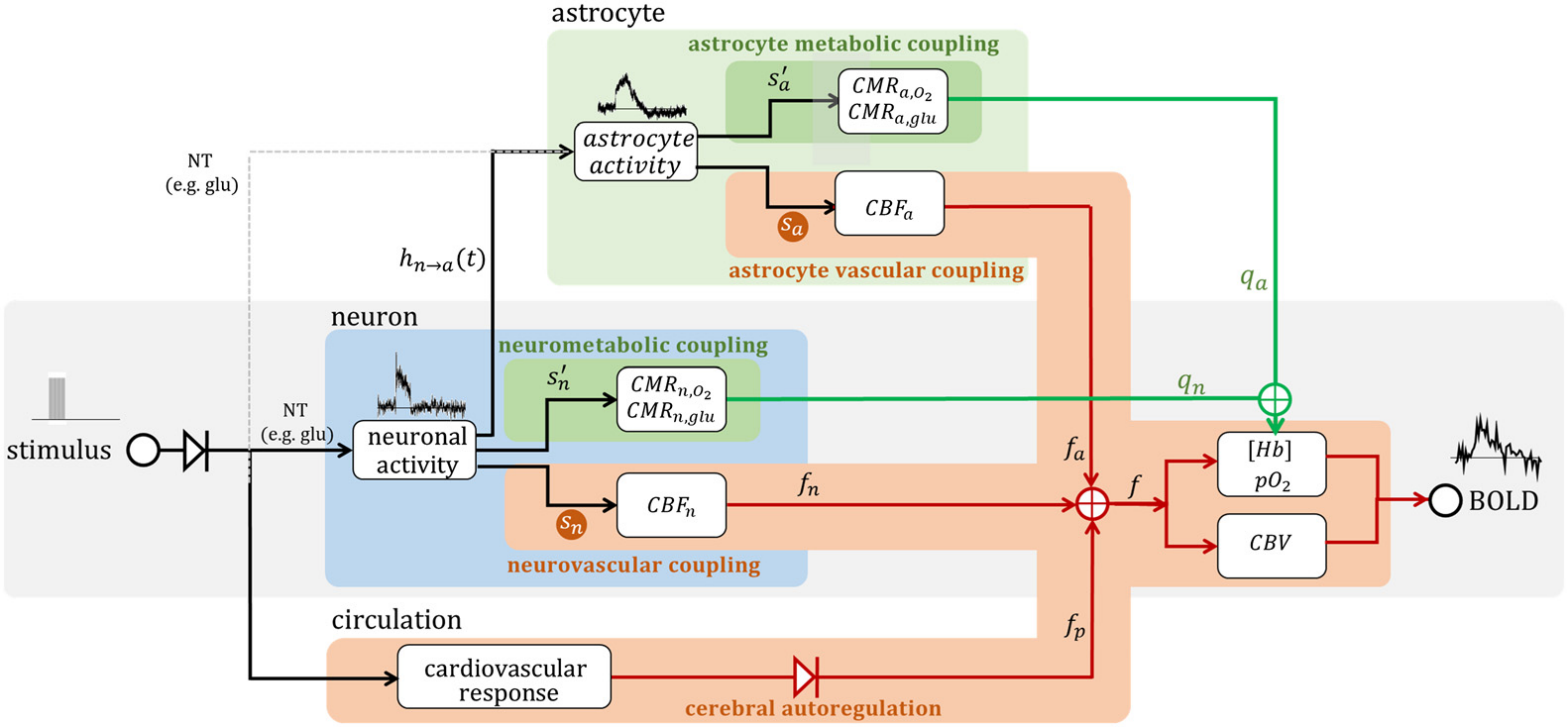
The canonical balloon model can be augmented to comprehensively account for signal modulation sources from the NVU. Depicted is an extended balloon model integration non-neuronal contributions to the BOLD signal with the originally proposed model (gray shading). The stimulus pulse train prompts both neuronal and astrocytic activation (as illustrated by the ${\mathrm{Ca}}^{2+}$ transient) which lead to a respective change in CBF ($f_{n}$, $f_{a}$) and changes in oxygen consumption ($q_{n}$, $q_{a}$). These effects are lumped into cell-type specific HRF (${\mathrm{HRF}}_{n}$, ${\mathrm{HRF}}_{a}$, see Fig. 4). In addition, stimulus-evoked changes in cardiac output (heart rate and/or blood pressure) may overrule cerebral autoregulation, prompting a nonspecific CBF response ($f_{p}$), which adds to the overall BOLD response.

The aim of noninvasive functional brain imaging is to provide an accurate estimate of the underlying neuronal activity. Employing optical recordings at multiple stages of NVC provides relevant information for optimizing forward modeling. To generate a more comprehensive picture of the observed BOLD response, we suggest extending the well-known balloon model, which predicts the changing venous blood volume and dHb content (thereby prompting the BOLD response), by including non-neuronal mechanisms shown to display stimulus-associated signal transients (Fig. 5). The original model (see, e.g., Ref. 11) depends only on the inflow of blood, with neuronal signaling (i.e., neurotransmitters acting directly on the vasculature) typically assumed as the only driving force. However, this fails to account for certain key features of the BOLD response, such as the prolonged response phase or poststimulus undershoot. Based on our previous research, we suggest two additional main sources associated with CBF changes: astrocytic activity^39^^,^^40^ leading to the release of vasoactive molecules, and depending on the physiological state potentially to altered cardiovascular activity causing a systemic change in blood flow that may overrule cerebral autoregulation. The last point is of particular relevance for studies using anesthetized animals. While the former two sources are part of intact neurovascular coupling, cardiovascular responses, such as stimulus-evoked sudden changes in heart rate and blood pressure,^117^ constitute a confounding factor that should be minimized.^118^^,^^119^

Animals studies revealed interference of anesthesia with CNS physiology at various levels. It was shown to affect neuronal excitability per se without affecting NVC,^120^ to compromise NVC,^121^ or to alter systemic cardiovascular output,^117^ or to act by a combination of these effects. In all these conditions, anesthesia would induce alterations in the stimulation induced FH, though the causes would be rather different, and correspondingly the relationship between neuronal signals and FH responses. Such limitations have to be considered when using results obtained from anesthetized animals for the interpretation of data obtained in conscious humans. Yet, a detailed discussion of these aspects is beyond the scope of this article.

A forward model of the neuronal-astrocytic-vascular ${\mathrm{Ca}}^{2+}$ signaling cascade has recently been shown to accurately reproduce BOLD responses.^122^ However, the assumption of astrocytes as a passive intermediary between neurons and the vasculature fails to account for situations where the neuronal activity (but not necessarily the astrocytic activity) and the BOLD response are uncoupled or even inverted.^68^^,^^108^ The hope of creating more accurate forward models is that, by inverting the model, accurate inferences about neuronal activity could be made solely based on the BOLD response. Despite the growing evidence that the BOLD signal is not driven by neuronal activity alone, refined forward models could help define the boundaries within which the NVC is intact, and thus prevent erroneous interpretations of BOLD fMRI.

## 4 Optical Signals for Neuronal Stimulation: Insights into Monoaminergic Systems

**Fig. 6:**
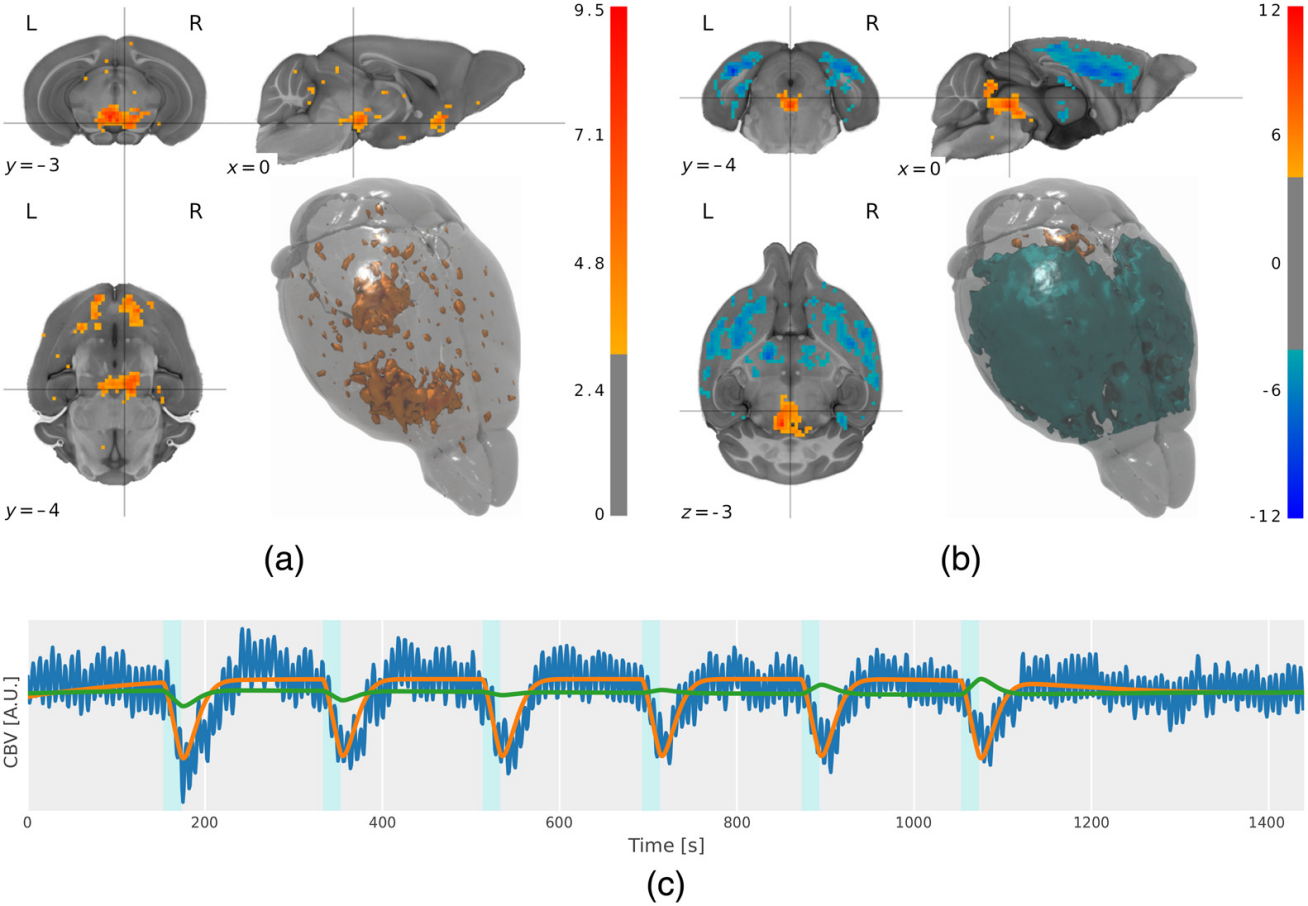
Opto-fMRI can be leveraged to image multimodal activity patterns elicited by widely projecting neuronal systems with low endogenous activity profiles. Optogenetics permits both neurotransmitter-specific selectivity, which can be used to target specific neuronal subpopulations, as well as high-amplitude signal enhancement, which can drive network population to sufficiently high levels of activity, as to be clearly modeled in time-resolved fMRI data. Depicted are (a), (b) both population-level activity maps, showing uniform and divergent valence of responses, respectively, as well as (c) a subject-level signal trace example. (a) Population-level t-statistical map of right VTA dopaminergic neuronal stimulation. Figure adapted from Ref. 75, with permission. (b) Population-level t-statistical map of dorsal raphe nucleus serotonergic neuronal stimulation. Figure adapted from Ref. 76, with permission. (c) Single-subject time course of mean signal from the dorsal raphe nucleus region of interest, during optogenetic stimulation of serotonergic neurons. The CBV signal trace is shown in blue, the response regressor (used to estimate the amplitudes mapped in (b) trace is shown in orange, and the response amplitude decay trace is shown in green. Figure adapted from reference analysis results of the SAMRI package.^123^^,^^124^

In preclinical neuropsychiatric applications, opto-fMRI has prominently been used to map the effective connectivity of neurotransmitter systems, as illustrated by the landmark results of mapping the dopaminergic^19^ and serotonergic^22^ systems [Figs. 6(a) and 6(b)]. On account of better statistical contrast, such mapping efforts produce more finely resolved spatial maps than corresponding chemogenetics approaches (cf. Refs. 22 and 125), albeit at the cost of increased invasiveness.

The reuse of these assays has demonstrated map reproducibility,^22^^,^^76^ qualitative translational consistency,^19^^,^^75^ as well as applicability to psychopharmacological profiling.^76^ Further, opto-fMRI has facilitated the disambiguation of dopaminergic and nondopaminergic ventral tegmental area (VTA) signaling in reward circuitry,^70^ and has uncovered significant deviations in functional dopaminergic VTA connectivity from what could linearly be inferred based on structural connectivity.^19^ A core advantage which renders such studies illuminating beyond simple SNR incrementation compared to chemogenetic or resting-state fMRI is that it constitutes a qualitative leap toward the *ceteris paribus* (i.e., “other things being held constant,” as commonly used to characterize the generalizability of statistical regularities.^126^) estimation of effective connectivity. Being able to drive neuronal subpopulations directly, the causal functional effects—rather than merely the correlative functional features—of activity in a system can be clearly highlighted (cf. “effective connectivity” and “functional connectivity”^127^). Although the limited temporal resolution of current fMRI technology renders an elucidation of second and higher order downstream signal relay infeasible, opto-fMRI is currently able to map out neuronal-subpopulation-based signal propagation one step at a time. Such stepwise functional projection profiles can further be conceptualized as phenotypical characteristics (i.e., functional neurophenotypes^128^) and be subject to experimental manipulation.

**Fig. 7:**
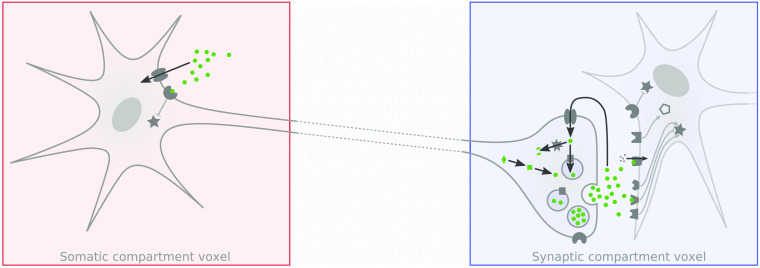
Opto-fMRI provides macroscopic resolution disambiguation of cell biological processes Depicted are neuronal schematics showing a somatic compartment and a synaptic compartment, as these may be seen in fMRI (distance between voxels not to scale). Depending on the statistical contrast of the stimulation as well as on the the neuronal system targeted, such different voxels may be more distant than the spatial autocorrelation range of fMRI—thus capturing potentially different responses in different cellular compartments. Such a difference may be seen in Fig. 6(b), where the red coded somatic voxel would correspond to the read heatmap voxels in the midbrain, and the blue coded voxel would correspond to the blue heatmap voxels in the cortex. The neuronal schematic showcases cell biological processes, such as neurotransmitter synthesis, anterograde synaptic transmission, autoinhibition, neurotransmitter reuptake, and degradation, laid out over cell compartments. Neurotransmitters and precursors are color-coded green and proteins involved in the aforementioned processes are coded gray. Figure adapted from Ref. 76, with permission.

A particular feature in the stimulation and whole-brain assay of long-ranging projections, including but not limited to monoaminergic projections, consists of the ability to differentiate responses across subcellular compartments at macroscopic resolutions—*in vivo* and noninvasively, with respect to the measurement modality. Given sufficiently high statistical contrast, combined with cell-type selective stimulation as achieved via opto-fMRI, neuronal compartments can be observed to show different response valences or kinetics. This is represented by a graphical model in Fig. 7, and substantiated by experimental results which showcase somatic and post/synaptic voxels displaying either similar [Fig. 6(a)] or opposite [Fig. 6(b)] responses. Hence, targeted activation of dopaminergic neurons in the VTA prompts postsynaptic excitation, while targeted activation of serotonergic neurons in the DR prompts postsynaptic inhibition.

Such localization is particularly relevant in the study of neuropsychiatric interventions, where targets may be differentially distributed across neurons (see receptor distributions in Fig. 7). Correspondingly, proof-of-principle applications of opto-fMRI have been able to deliver cell-compartment level profiling of both acute^22^ and chronic antidepressant action and have shown differential longitudinal profiles for somatic, cortical synaptic, and subcortical synaptic areas.^76^

On account of being neuronal subpopulation-selective as well as contingent on the induction of signal amplitudes sufficiently high to overcome the SNR constraints of fMRI, opto-fMRI commonly elicits neurotransmitter depletion. This manifests itself in incremental response-amplitude reduction (i.e., biologically meaningful refractory effects), as shown in Fig. 6(c). Though extant literature commonly handles this feature as an ancillary nuisance process, the ubiquity of this phenomenon constitutes a representative example for an implicit assay of a neurophysiologically relevant process, which can be reexamined in refined analysis instantiations. More generally, given the spatial standardization presupposed by whole-brain imaging, opto-fMRI characteristically produces manifold possibilities for quantitative data reuse and integration, as otherwise uncommon in the study of cell biological assays.

## 5 Conclusion

Recent advances in the development of hybrid optical/fMRI systems enabled tackling difficult research questions using the best of two worlds: The capabilities of fMRI to record brain functional parameters, and the plethora of genetic tools in optical neuroscience to investigate the role of individual cell types. Integrating signals at different spatial scales along the neurovascular pathway opens avenues to validate forward models of hemodynamic readouts. The usage of cell type-specific stimulation lends the capacity to bypass sensory signal input mechanisms and drive individual network nodes in the brain. Both in stimulation and complementary spatial scale measurements, correlations with cell biological phenomena enhance the causal understanding of neuronal function.

Such insights facilitate both conceptual advances in the accurate interpretation of fMRI data—thereby reflecting on extant results spanning a breadth of human and animal studies—as well as a stark increase in scope for fMRI capabilities.
